# Comprehensive Proteomic Analysis of Colon Cancer Tissue Revealed the Reason for the Worse Prognosis of Right-Sided Colon Cancer and Mucinous Colon Cancer at the Protein Level

**DOI:** 10.3390/curroncol28050305

**Published:** 2021-09-15

**Authors:** Yanyu Chen, Wenyun Hou, Miner Zhong, Bin Wu

**Affiliations:** 1 State Key Laboratory of Medical Molecular Biology & Department of Immunology, Institute of Basic Medical Sciences, Chinese Academy of Medical Sciences, Beijing 100730, China; chenyanyu0527@126.com; 2 Department of General Surgery, Peking Union Medical College Hospital, Chinese Academy of Medical Sciences and Peking Union Medical College, Beijing 100730, China; pumc_hwy@student.pumc.edu.cn (W.H.); zhongmer@mail.sysu.edu.cn (M.Z.)

**Keywords:** colon cancer, mucinous carcinoma, proteomics, tumor location

## Abstract

To clarify the molecular mechanisms underlying the poor prognosis of right-sided and mucinous colon cancer at the proteomic level. A tandem mass tag-proteomics approach was used to identify differentially expressed proteins (DEPs) in colon carcinoma tissues from different locations and with different histological types to reveal the underlying mechanisms of these differences at the protein level. In additional, the DEPs were analyzed using bioinformatics methods. The proteomics profiles among colon cancers with different tumor locations and histological types were dramatically distinguished. In terms of tumor locations, the right-sided carcinoma specific DEPs may promote the tumor progression via activating inflammation, metastasis associated pathways. When referring to histological types, the mucinous colon cancers perhaps increased the invasion and metastasis through distinct mechanisms in different tumor locations. For mucinous cancer located in right-sided colon, the mucinous specific DEPs were mainly associated with ECM-related remodeling and the IL-17 signal pathway. For mucinous cancer located in left-sided colon, the mucinous specific DEPs showed a strong relationship with ACE2/Ang-(1–7)/MasR axis. The proteomics profiles of colon cancers showed distinct differences related to locations and histological types. These results suggested a distinct mechanism underlying the diverse subtypes of colon cancers.

## 1. Introduction

Colorectal cancer (CRC) is the second leading cause of cancer-related death and the fourth most frequently diagnosed cancer worldwide [1]. In recent years, with improvements in precise and individualized treatments, there is a pressing requirement for the refinement of the classification of CRC subtypes based on their tumor locations and histological characteristics. Multiple studies have indicated that the clinical behavior of CRC differs with the different locations and histological types, suggesting a distinct mechanism underlying the different CRC subtypes [2,3,4,5].

According to the location of the primary tumor, CRC can be divided into left-sided CRC in the splenic flexure, descending colon, sigmoid colon and rectum, and right-sided CRC in the region from the liver flexure to the cecum [6]. A retrospective analysis showed that the location of the primary tumor can be used to predict the treatment outcome and metastasis of colon cancer [2]. Additionally, the prognosis of right-sided tumors was found to be worse than that of left-sided tumor [7]. In another retrospective study of 4034 patients with stage III colon cancer, patients with right-sided tumors had shorter overall survival than those with left-sided tumors [8]. A more comprehensive comparison and evaluation of the differences between left-sided and right-sided colon cancer is required to improve the treatment of colon cancer.

Mucinous carcinoma, a distinct subtype of CRC, is characterized by abundant mucin that constitutes >50% of the tumor volume [9]. Compared with adenocarcinoma, mucinous carcinoma is more often diagnosed in an advanced stage [10]. In particular, it has been reported that mucinous carcinoma has a different molecular signature that might cause faster disease progression [11]. Multiple studies have suggested that the mucinous type of colorectal carcinoma is generally an unfavorable prognostic indicator and less responsive to chemotherapy compared to non-mucinous colorectal carcinoma [12,13,14]. In several clinical trials, mucinous colorectal adenocarcinoma patients showed a significantly inferior median overall survival (OS) than non-mucinous colorectal adenocarcinoma patients [13,15,16].

Therefore, in this study, we used high-throughput quantitative protein mass spectrometry (MS) to compare the protein expression profiles of left-sided mucinous colon cancer (LMC) tissue, right-sided mucinous colon cancer (RMC) tissue, left-sided non-mucinous colon cancer (LNMC), right-sided non-mucinous colon cancer (RNMC) tissue, and normal colonic (NC) tissue. By comparing left-sided versus right-sided and mucinous versus non-mucinous colon cancer tissues, we identified the crucial differentially expressed proteins (DEPs) using bioinformatics analysis to reveal the underlying mechanisms of the differences at the protein level.

## 2. Materials and Methods

### 2.1. Patients

A total of 29 colon cancer patients were recruited at the Peking Union Medical College Hospital (PUMCH, Beijing, China) from 2015 to 2017. The following exclusion criteria were applied: (a) with diabetes, autoimmune diseases, or blood diseases; (b) with a history of chemo- or radiotherapy, or a previous history of malignancy; (c) transverse colon cancer; and (d) rectal cancer. The normal colon tissues of 25 patients were included in the NC group; cancer tissues from six patients with left-sided mucinous colon cancer were included in the LMC group; cancer tissues from eight patients with left-sided non-mucinous colon cancer were included in the LNMC group; cancer tissues from seven patients with right-sided mucinous colon cancer were included in the RMC group; and cancer tissues from eight patients with right-sided mucinous colon cancer were included in the RNMC group. See Table S1 for details. This study was approved by the Ethics Committee of PUMCH (No. S-k655).

### 2.2. Tandem Mass Tag (TMT) Labeling

The colon tissue obtained in each case was homogenized in an ice-cold lysis buffer (8 M urea in phosphate-buffered saline [PBS], pH 8.0, 1× cocktail, 1 mM phenylmethanesulfonyl fluoride [PMSF]) using a Q800R3 sonicator (Qsonica, Newtown, CT, USA). After centrifugation at 12,000 rpm for 15 min at 4 °C, the supernatant was transferred to a fresh tube, and the protein concentration was determined using a Nanodrop 2000 (Thermo Scientific, Branchburg, NJ, USA) according to the manufacturer’s instructions. The poteins were reduced, digested, and labeled using TMT reagents. Different colon tissues were labeled with the following tags: TMT-126 for NC, TMT-128 for LMC, TMT-130 for LNMC, TMT-129 for RMC, and TMT-131 for RNMC.

### 2.3. HPLC and LC-MS/MS Analysis

TMT-labeled peptides were loaded onto an Xbridge BEH300 C18 column (4.6 × 250 mm^2^, packed with 5 µm, 300 Å resin, Waters, Milford, MA, USA) controlled by the UltiMate 3000 HPLC workstation (Thermo Scientific, Branchburg, NJ, USA). The peptides were eluted using a gradient elution buffer consisting of H_2_O (pH 10.0) and acetonitrile (pH 10.0) at a flow rate of 1.0 mL/min. Fractions were collected every 1.5 min into 47 tubes, and then dried and combined in 12 tubes in a Speedvac concentrator. Finally, the samples were dissolved in 20 µL 0.1% trifluoroacetic acid (TFA) for subsequent liquid chromatography (LC)–MS/MS analysis.

The dissolved fractions were analyzed using the Lumos mass spectrometer as described previously. Briefly, the fractions were separated by a 135 min gradient elution (Phase A: H_2_O with 0.1% formic acid; Phase B: 100 acetonitrile and 0.1% formic acid) at a flow rate of 0.3 µL/min with the UltiMate 3000 RSLCnano System (Thermo Scientific, Branchburg, NJ, USA) interfaced with the Thermo Orbitrap Fusion Lumos mass spectrometer. A fused-silica capillary column (75 µm ID, 150 mm length; Upchurch, Oak Harbor, WA, USA) packed with C18 resin (300 Å, 2 µm; Varian, Lexington, MA, USA) was used as the analytical column. The Lumos mass spectrometer was operated in the data-dependent acquisition mode using Xcalibur 4.1 software (Thermo Scientific, Waltham, MA, USA). Using Orbitrap (350–1550 *m*/*z*, 120,000 resolution), a single full-scan mass spectrum was performed followed by 3-s data-dependent MS/MS scans at 35% normalized collision energy (higher energy C-trap dissociation, HCD).

### 2.4. Protein Identification Using MS/MS Data

Mass spectra raw data were then analyzed using the Proteome Discoverer 2.2 software (Thermo Scientific, Waltham, MA, USA) by automatically searching against the reviewed UniProt Swiss-Prot FASTA database (released on 5 February 2018). The following search criteria were applied: a maximum number of two missed trypsin/Lys-C cleavages allowed; mass tolerance for precursor and fragment ions set at 10 ppm (all MS in an Orbitrap mass analyzer) and 0.02 Da (all MS2 spectra); carbamidomethylation (C, +57.021 Da) and TMT-6plex (K and peptide N-terminus) set as static modifications; and oxidation (methionine, M) specified as the dynamic modification. The proteins and peptides identified within a 1% global false discovery rate were selected for further analysis. Based on the representative MS/MS spectral data of identified peptides and the intensity of TMT precursors, relative protein intensities were quantified using the TMT-6plex method. Details of the MS proteomics data can be obtained from the ProteomeXChange Consortium (http://www.proteomexchange.org, accessed on 4 April 2021) via the Pride Partner Repository (dataset identifier PXD022802).

### 2.5. Bioinformatics Analysis

For proteomic analysis of colon cancer tissues, a 1.5-fold change was set as the threshold for differential expression of proteins [17,18]. Principal component analysis (PCA) and Pearson correlation analysis were conducted using packages in the R program (R Foundation for Statistical Computing, Vienna, Austria. http://www.R-project.org/, 21 November 2020). Scatter plots and hierarchical cluster analyses of DEPs were carried out using the JMP Pro (version 13.0, SAS Institute, Cary, NC, USA). Gene Ontology (GO) functional enrichment analysis, and Kyoto Encyclopedia of Genes and Genomes (KEGG) pathway analysis were performed using R program by the clusterProfiler package and Cytoscape plug-in ClueGO. Protein–protein interaction (PPI) analysis was performed and visualized using the string APP plugin in Cytoscape (Version 3.7.1). GEPIA (http://gepia.cancer-pku.cn/, 12 April 2021) online database was used to validate clinicopathological and prognostic information of the hub genes in the cancer genome atlas (TCGA).

## 3. Results

### 3.1. Differences in Colon Cancer Location and Histological Type Were Related to Distinct Proteomic Profiles

**Figure 1:**
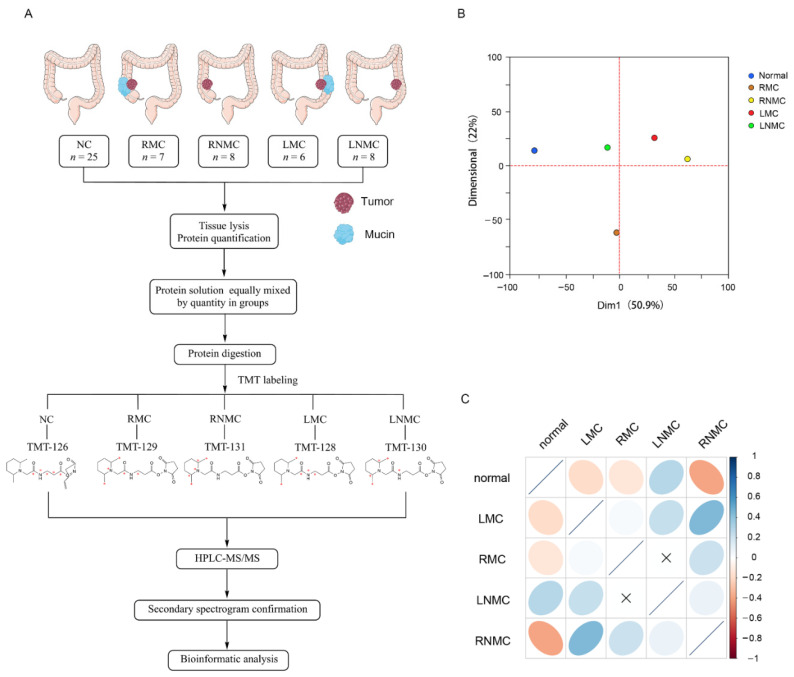
Quantitative characteristics of protein profiles. (**A**) Experimental workflow. (**B**) Principal component analysis of protein profiles of normal, RMC, RNMC, LMC, and LNMC. (**C**) Correlation matrix showing the protein abundance correlations between normal, RMC, RNMC, LMC, and LNMC.

It is well known that the clinical behavior of colon cancer differs with the location and histological type. Here, we compared protein abundances between group RMC and LMC, RMC and RNMC, LMC and LNMC, to obtain a comprehensive description of colon cancer DEPs associated with different sites and histological types. The protein composition of NC, RMC, LMC, LNMC and RNMC tissues was determined using TMT-based quantitative MS technology (Figure 1A). The MS raw data were processed by Proteome Discoverer (version 2.2 Thermo Scientific, Waltham, MA, USA). In total, 5621 credible proteins were identified. To avoid contamination, after excluding high-abundance serum proteins, a total of 5594 proteins were included in the follow-up analysis (Table S2).

PCA was used to assess similarities between the groups and revealed differences in the proteome profiles of NC, RMC, LNMC, RNMC, and LMC tissues (Figure 1B). PCA showed clear and equal separation of the five samples.

Next, we performed a simple correlation analysis to investigate potential relationships among the five groups (Figure 1C). Intriguingly, we discovered very low correlations between RMC and LMC and between RNMC and LNMC. This indicated a low level of similarity in the protein expression profiles of colon cancer in different tumor locations. Since the groups of same histologic type but different tumor locations were poorly correlated, we hypothesized that tumor location has a significant impact on the protein expression in colon cancer. Therefore, we compared the abundance of proteins between RMC and RNMC and between LMC and LNMC.

### 3.2. Functional Analysis of DEPs between RNMC and LNMC

**Figure 2:**
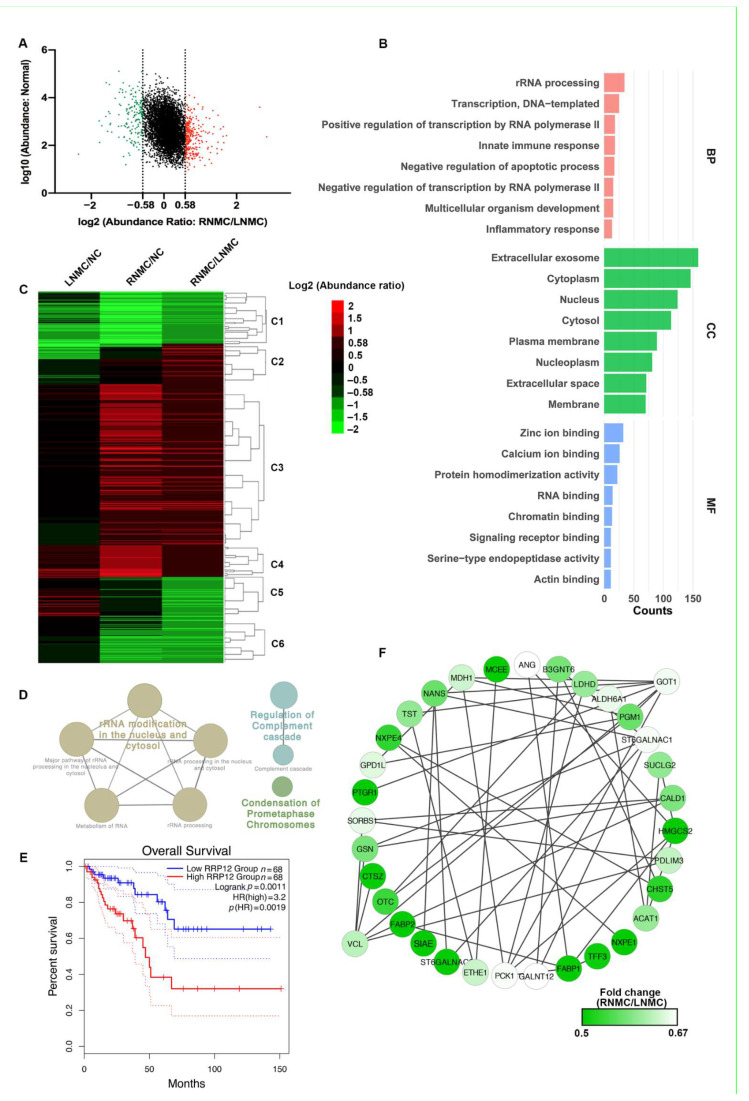
Functional analysis of differentially expressed proteins (DEPs) between RNMC and LNMC tissues. (**A**) Scatter plot showing the distribution of upregulated (red dots) and downregulated (green dots) DEPs. (**B**) GO analysis indicated enrichment of these DEPs in biological processing (BP), cellular component (CC), and molecular function (MF). (**C**) Hierarchical clustering analysis and heatmap of DEPs. The heatmap was constructed based on a log2 transformation of relative abundance ratios (RNMC/LNMC). (**D**) REACTOME pathway analysis of the RNMC-specific upregulated DEPs. (**E**) The Kaplan–Meier survival analysis indicated inferior overall survival among colon cancer patients with high RRP12 expression. (**F**) The RNMC-specific downregulated DEPs in the protein–protein interaction networks are shown as nodes (MS data presented as the 131/130 ratios were matched to STRING networks). The intensity of the green color indicates the ratio of protein abundance in RNMC/LNMC. The protein abundance of tumor tissue relative to normal tissue is indicated in different colors, with red indicating up-regulated protein and green indicating down-regulated protein.

Using a 1.5-fold change cut-off for the classification of differential expression, we identified a total of 456 (286 upregulated and 170 downregulated) DEPs in RNMC versus LNMC (Figure 2A). GO analysis indicated that these DEPs participated mainly in the biological processes of rRNA processing, and negative regulation of apoptotic process and inflammatory response were related to the cellular components of cytoplasm, nucleus, and extracellular space and performed molecular functions of zinc iron binding, protein homodimerization activity, and RNA binding (Figure 2B).

To demonstrate the differences in protein expression levels between RNMC and LNMC, hierarchical cluster analysis was carried out and a heatmap was generated (Figure 2C). The information for the proteins in each cluster is shown in Table S3. Using normal tissues as the control group, we compared the expression of 456 DEPs in RNMC and LNMC. In cluster 3 (C3), 202 RNMC-specific DEPs were upregulated, and in cluster 6 (C6), 65 RNMC-specific DEPs were downregulated (Figure 2C).

REATOME pathway analysis revealed that the RNMC-specific upregulated DEPs were enriched in three types of pathways: rRNA processing, regulation of complement cascade, and condensation of prometaphase chromosomes (Figure 2D). REACTOME pathway analysis of the RNMC-specific upregulated DEPs was shown in Table S4. We identified 28 RNA-processing-related proteins, including U3 small nucleolar RNA-associated proteins UTP3, UTP14A, UTP11, and ribosomal RNA-processing proteins RRP1, RRP7A, and RRP12. Among these RNMC-specific upregulated proteins, RRP12-like protein (RRP12), was positively correlated with poor prognosis of patients with colon cancer. The Kaplan–Meier survival analysis using GEPIA (http://gepia.cancer-pku.cn/, accessed on 12 April 2021) [19] online tool based on TCGA database indicated inferior OS among colon cancer patients with high RRP12 expression (Figure 2E).

In addition to the RNMC-specific upregulated DEPs, we also analyzed the interactions of these RNMC-specific downregulated DEPs using STRING online resource. PPI networks were visualized by Cytoscape based on their STRING fractions. The complex network of PPIs is shown in Figure 2F. Glutamate oxaloacetate transaminase 1 (GOT1) and vinculin (VCL) were identified as the central proteins in this integrated protein network.

### 3.3. RNMC-Specific Upregulated DEPs Enriched in Inflammation-, Metastasis- and Proliferation-Associated Pathways

Pathway analysis of RNMC-specific DEPs revealed that they were mainly enriched in the complement activation pathway, epithelial-mesenchymal transition (EMT) in the CRC pathway and the mitogen-activated protein kinase (MAPK) signaling pathway, which are related to cell proliferation, differentiation, inflammation and EMT. These pathways were visualized using Cytoscape software, and the protein abundance in LNMC and RNMC were compared with that in NC.

**Figure 3:**
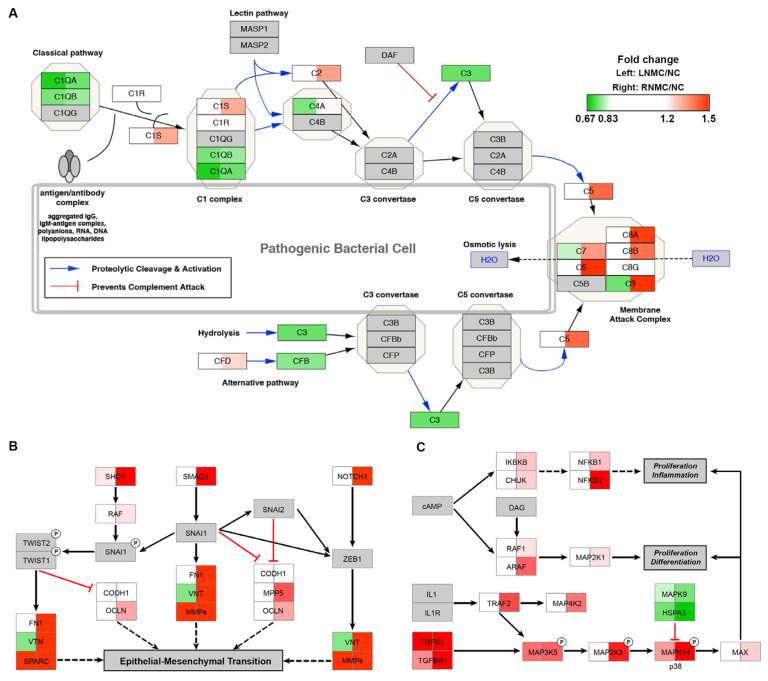
Pathways enriched in the RNMC-specific DEPs. MS data presented as the 130/126 (left) and 131/126 (right) ratios. Up or downregulation of identified proteins is indicated by colors in the pathway (upregulated in red, downregulated in green). (**A**) The DEPs mapped to the complement activation pathway. (**B**) The DEPs mapped to EMT in the CRC pathway. (**C**) The DEPs mapped to the MAPK signaling pathway. The protein abundance of tumor tissue relative to normal tissue is indicated in different colors, with red indicating up-regulated protein and green indicating down-regulated protein.

In the RNMC versus NC comparison, C1s, C2, C5, and multiple components of the membrane attack complex, including C6, C7, C8A, C8B, and C9, in the complement activation pathway, which is related to inflammation, were significantly upregulated in RNMC (Figure 3A). In the EMT in the CRC pathway, proteins such as fibronectin (FN1), vitronectin (VTN), mothers against decapentaplegic homolog 2 (Smad2), neurogenic locus notch homolog protein 1 (Notch1), MAGUK p55 subfamily member 5 (MPP5), and SHC transforming protein 1 (SHC1), which promoted EMT, were all specifically upregulated in RNMC (Figure 3B). Cell proliferation, differentiation, and inflammation can be promoted via the MAPK signaling pathway. Proteins involved in the MAPK pathway activation were upregulated in RNMC, while proteins that inhibit MAPK pathway activation were downregulated. Some of these proteins, such as NFKB1, NFKB2, and MAP3K2 were identified as RNMC-specific DEPs. Although other DEPs, such as TGFBR1, MAPK14, and MAPK9, changed in the same direction as LNMC and RNMC, the degree of change was greater in RNMC (Figure 3C).

### 3.4. GO Analysis Revealed That RMC Specific DEPs Were Involved in the ECM Structure Proteins and Associated with Poor Prognosis

**Figure 4:**
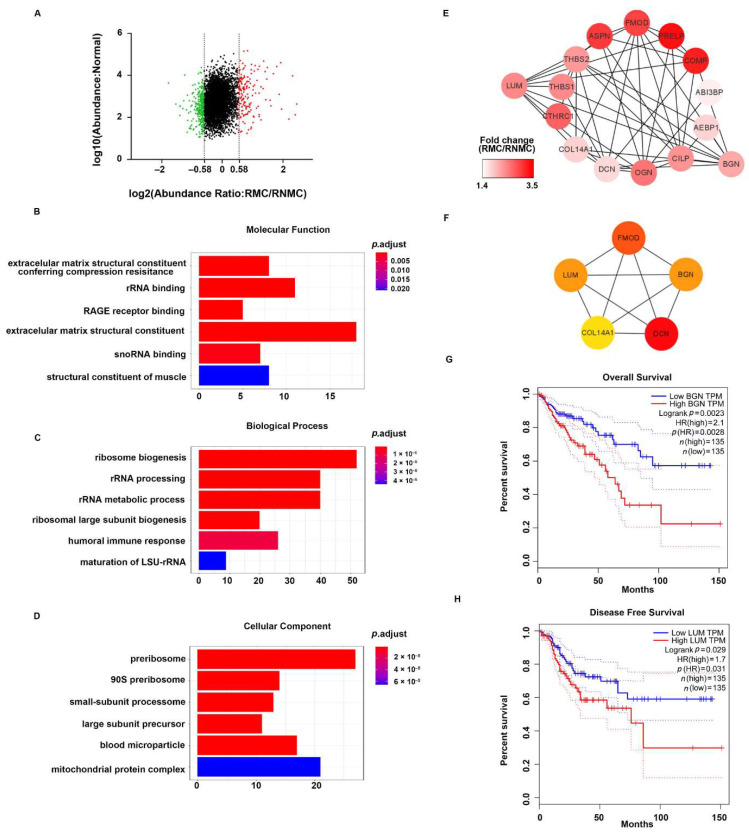
Functional analysis of DEPs between RMC and RNMC. (**A**) Scatter plot showing the distribution of upregulated (red dots) and downregulated (green dots) differentially expressed proteins (DEPs). (**B**–**D**) GO analysis indicated enrichment of these DEPs in molecular function (MF), biological processing (BP), and biological cellular component (CC). (**E**) The ECM-related DEPs in the protein–protein interaction networks are shown as nodes (MS data presented as the 129/131 ratios were matched to STRING networks). The intensity of the red color indicates the ratio of protein abundance in RMC/RNMC. (**F**) The hub proteins of ECM-related DEPs identified by the MCODE plug-in in RMC. The Kaplan–Meier survival analysis indicated inferior overall survival and disease-free survival among colon cancer patients with high expression of BGN and LUM, respectively. (**G**) The elevated BGN mRNA expression in colorectal cancer is positively associated with poor prognosis (log-rank test *p* = 0.0023). (**H**) The elevated LUM mRNA expression in colorectal tumor tissues is also positively associated with poor prognosis (log-rank test *p* = 0.029).

To explore the significance of mucinous differentiation in right-sided colon cancer, we compared the differences in protein expression between RMC and RNMC. A total of 444 DEPs were identified in the comparison of RMC with RNMC consisting of 156 upregulated proteins (protein relative abundances > 1.5) and 288 downregulated proteins (protein relative abundances < 0.67) (Figure 4A). See Table S5 for details.

To provide an overview of the constitution of the proteome in right-sided colon cancer, we clustered all the identified DEPs into GO categories using the ClusterProfiler package in R (R Foundation for Statistical Computing, Vienna, Austria). Molecular function analysis revealed that these proteins were mainly extracellular matrix (ECM) structural constituents (Figure 4B). In addition, the DEPS related to biological process showed a significant enrichment in the categories of ribosome biogenesis and rRNA processing (Figure 4C). Cellular component analysis revealed that the proteins were mostly enriched in the categories of pre-ribosome, small-subunit processome, and large-subunit processome (Figure 4D). These results suggested that ECM structure proteins play more important roles in RMC than in RNMC.

To gain further insights into the ECM-related DEPs in RMC, we constructed a comprehensive protein network using Cytoscape 3.7.1 to visualize the potential relationships between these proteins. The mapping revealed that almost all the identified proteins were upregulated. STRING PPI analysis showed close interactions among these upregulated proteins (Figure 4E). In addition, hub genes in this network were ranked using the CytoHubba plug-in in Cytoscape. The top five hub proteins were FMOD, LUM, BGN, COL14A1, and DCN, indicating that these five genes play essential roles in the ECM (Figure 4F). Then, we used the online Gene Expression Profiling Interactive Analysis (GEPIA) tool to evaluate the prognostic value of the five hub genes in TCGA database. We found that the group with high BGN expression showed significantly decreased overall survival (OS) compared with the low BGN expression group (*p* = 0.0023, Figure 4G). LUM showed a negative correlation for disease-free survival (DFS) prognostic ability in the TCGA database (*p* = 0.029, Figure 4H).

### 3.5. Pathway Analysis of DEPs between RMC and RNMC Enriched in IL-17 Signal Pathway

**Figure 5:**
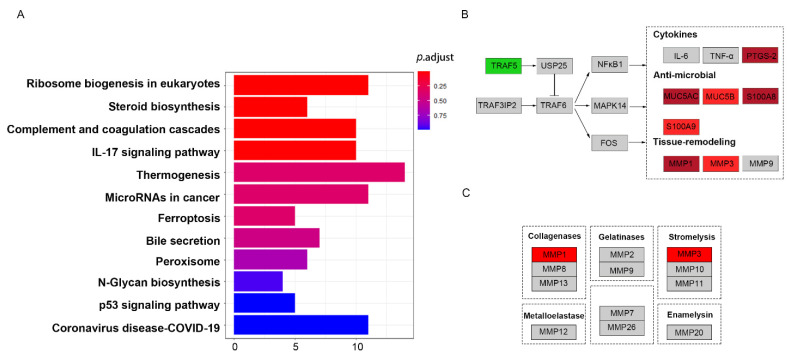
Pathway analysis of DEPs between RMC and RNMC. The identified proteins were indicated by colors in the pathway (upregulated in red, downregulated in green). (**A**) The DEPs enriched in the KEGG pathways. (**B**) The DEPs mapped to the IL-17 pathway. (**C**) The DEPs mapped to the matrix metalloproteinase signaling pathway.

To gain a better understanding into the biological pathways in RMC, KEGG pathway analysis was performed using the ClusterProfiler package in R. The results revealed that ribosome biogenesis in eukaryotes, steroid biosynthesis, complement and coagulation cascades, and the IL-17 signaling pathway were the most over-represented among the DEPs (Figure 5A). These pathways were related to tumor proliferation and progression. These DEPS in matched IL-17 signaling pathways based on the KEGG pathway database were visualized using the KEGGParser plug-in in Cytoscape (version 3.7.1). Many DEPs are known to be involved in the gel-forming mucin that is thought to contribute to lubricating properties. The DEPS enriched in the IL-17 signaling pathway were also consistent with known mucinous tissue proteins. As shown in Figure 5B, the IL-17 signaling pathway mapped with DEPs in RMC relative to RNMC. Mapping revealed that most of the identified proteins were upregulated, except TRAF5. Among the proteins implicated in the IL-17 signaling pathway, PTGS-2, MUC5AC, MUC5B, S100A8, and S100A9 were all upregulated. These results indicated that the DEPs in RMC were strongly related to the IL-17 signaling pathway. In addition, the DEPs mapped to the IL-17 signaling pathway were perhaps upregulated as a result of activation of the NF-κB and MAPK signaling pathways. MMP-1 and MMP-3, both matrix metalloproteinases, were identified among the DEPs. Mapping of the gene symbol of the DEPs to the matrix metalloproteinase pathway in the Wiki pathway database (Figure 5C) revealed that upregulation of MMP-1 and MMP-3 was related to collagenases and stromelysins, respectively, which play vital roles in ECM remodeling. MMPs are widely associated with features of cancer pathology, including invasion, metastasis, and angiogenesis.

### 3.6. GO Analysis Revealed That LMC Specific DEPs Were Enriched in the Renin-Angiotensin System and Control the Angiotensin Levels

**Figure 6:**
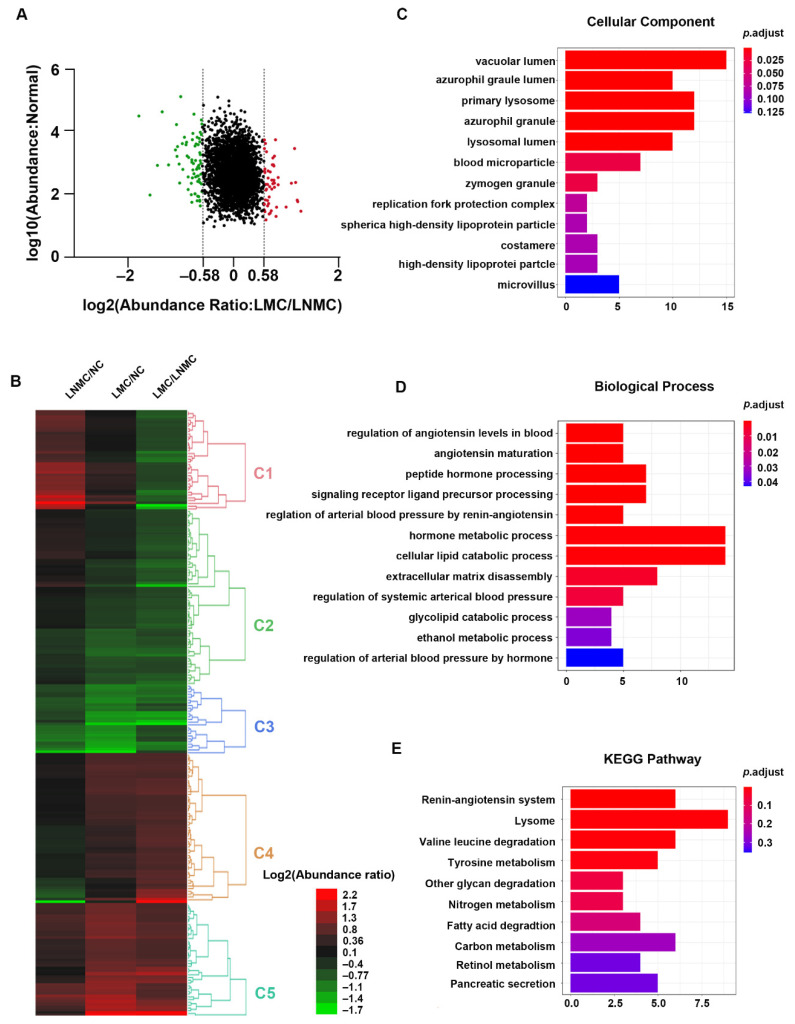
Functional analysis of DEPs between LMC and LNMC. (**A**) Scatter plot showing the distribution of upregulated (red dots) and downregulated (green dots) DEPs. (**B**) Hierarchical clustering analysis and heatmap of DEPs. The heatmap was constructed based on a log2 transformation of relative abundance ratios (LMC/LNMC). (**C**,**D**) GO analysis indicated the enrichment of these DEPs in cellular component (CC) and biological processing (BP). (**E**) Pathway analysis of DEPs between LMC and LNMC.

Left-sided colon cancer is considered biologically different from right-sided colon cancer. Furthermore, we speculate that mucinous histologic type colon cancer results in poor prognosis through a different mechanism in LCC compared with RCC. A total of 219 proteins were identified as DEPs in the comparison of LMC with LNMC. Among the 219 DEPs, 95 were upregulated (protein relative abundances > 1.5) and 124 were downregulated (protein relative abundances < 0.67), and the heatmap is shown in Figure 6A,B. The DEPs were grouped into five clusters (Clusters 1–5) based on the TMT-labeled protein ratios in different tissue types. As shown in Figure 6, the DEPs in Cluster 1 were downregulated in LMC relative to LNMC, but unchanged in LMC relative to NC. The DEPs in Clusters 2 and 3 were downregulated in LMC relative to LNMC and in LMC relative to NC. The DEPs in Clusters 4 and 5 were both upregulated in LMC relative to LNMC and in LMC relative to NC (Table S6).

GO enrichment analysis was performed on all the DEPs in an in-depth investigation of the distinct molecular mechanisms related to LMC relative to LNMC. GO cellular component analysis revealed that the majority of DEPs were located in the vacuolar lumen, azurophil granule lumen, primary lysosome, and bold microparticles. In the biological process analysis, the proteins were enriched in the categories of regulation of angiotensin levels in blood, angiotensin maturation, peptide hormone processing, and regulation of arterial blood pressure by renin-angiotensin (Figure 6C,D). To provide a glimpse into the biological pathways, KEGG pathway analysis was performed with ClusterProfiler package in R. The results showed that the DEPs were enriched in pathways related to the renin-angiotensin system; lysosome, valine, and leucine degradation; tyrosine metabolism; and degradation of other glycans (Figure 6E). These enriched pathways were consistent with those in the biological process category, which revealed that the DEPs were enriched in regulation of angiotensin levels in the blood. The renin-angiotensin pathway plays a crucial role in cancer biology and affects tumor growth by remodeling the tumor microenvironment.

### 3.7. Pthway Analysis of DEPs between LMC and LNMC Related to Renin-Angiotensin Pathway

**Figure 7:**
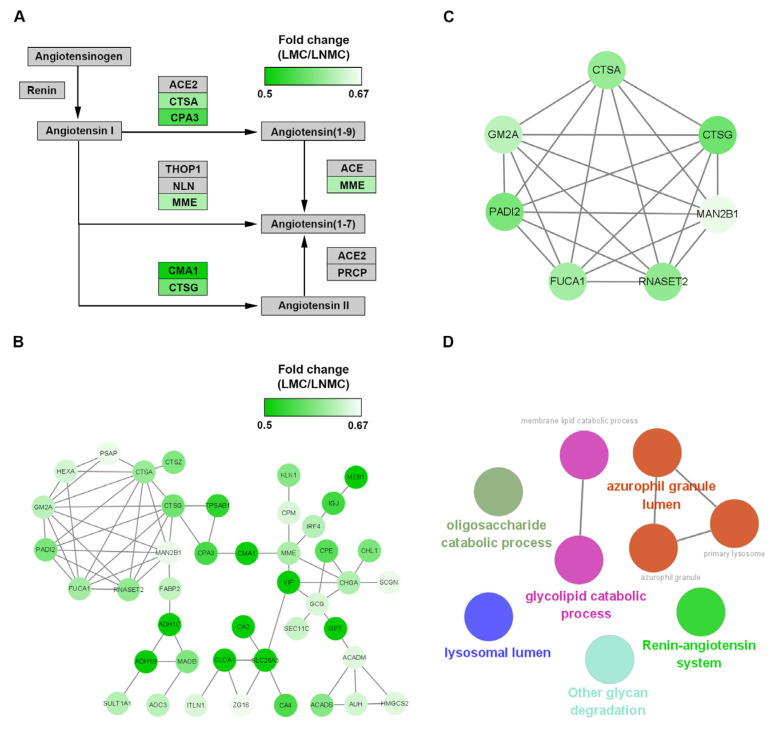
Pathways and protein–protein interaction network analysis of downregulated differentially expressed proteins (DEPs) in LMC. (**A**) KEGG pathway analysis of the LMC-specific downregulated DEPs. (**B**) The LMC-specific downregulated DEPs in the protein–protein interaction networks are shown as nodes (MS data presented as 128/130 ratios were matched to STRING networks). The depth of the green color indicates the ratio of protein abundance in LMC/LNMC. (**C**) The downregulated hub genes identified by the MCODE plug-in. (**D**) Functional analysis of the seven hub DEPs using the ClueGO plug-in by Cytoscape.

The DEPs enriched in the renin-angiotensin pathway were also visualized using Cytoscape (Figure 7A). The expression ratios of the DEPs between LMC and LNMC were represented by color intensity in heatmaps. Among the mapped proteins, lysosomal protective protein (CTSA), cathepsin G (CTSG), mast cell carboxypeptidase A (CPA3), neprilysin (MME), and calmodulin-1 (CMA1) were all strongly downregulated (relative abundance of protein as a TMT-128/TMT-130 ratio < 0.67). The pathway analysis indicated that LMC was closely associated with the angiotensin pathways. As show in Figure 7A, the downregulated DEPs mapped to the pathway had the capacity to inhibit the formation of angiotensin (1–7), which is related to tumor growth, invasion, and metastasis in various cancers.

To gain insights into the downregulated DEPs in the context of known PPIs, we investigated PPIs between proteins in Clusters 2 and 3 using the online tool STRING. PPI networks were constructed and visualized by using Cytoscape (Figure 7B). The hub proteins were identified by the molecular complex detection (MCODE) plug-in from the PPI network. Finally, six core proteins (CTSA, CTSG, GM2A, PADI2, MAN2B1, and RNASET2) were identified in MCODE 1 (Figure 7C). These core proteins were further analyzed using the ClueGO plug-in in Cytoscape to determine the biological function of the MCODE 1 module. In this analysis, the six proteins were related mainly to the renin-angiotensin system, lysosomal lumen, azurophil granule lumen, glycolipid catabolic process, and oligosaccharide catabolic process (Figure 7D).

## 4. Discussion

While differences in right-sided versus left-sided and mucinous versus non-mucinous colon cancer have been widely investigated, differences in protein levels between mucinous and non-mucinous colon cancer in different tumor locations have not yet been investigated using proteomics techniques. In this study, we investigated the molecular mechanism responsible for the differences in colon cancer in different locations and histological types. We conducted a TMT-based proteomics analysis to produce a comprehensive description of the changes in colon cancer protein profiles with different tumor locations and histological types.

### 4.1. RNMC-Specific DEPs Play an Important Role in the Progression of RNMC Compared with LNMC

As a hub protein among the RNMC-specific downregulated DEPs, GOT1 is an important regulator of glutamate levels, with inconsistent roles in different tumors. GOT1 overexpression was shown to promote cisplatin resistance in NSCLC [20]. In acute myeloid leukemia, upregulation of GOT1 was also found to be associated with a worse prognosis [21]. However, in cervical cancer, upregulation of GOT1 mRNA is a protective factor, indicating a better prognosis [22]. In pancreatic ductal carcinoma, GOT1 knockdown abolished glycolysis, nucleotide metabolism and redox homeostasis, which inhibited tumor growth, while this was not seen in CRC [23]. In this study, GOT1 was specifically downregulated in RNMC and interacted with multiple RNMC-specific downregulated DEPs, which may be related to the poor prognosis of RNMC. However, the mechanism remains to be fully elucidated.

VCL, another hub protein among the RNMC-specific downregulated DEPs, is an F-actin binding protein involved in cell matrix adhesion and intercellular adhesion [24]. Oestrogen receptor alpha suppressed cell amoeboid-like movement by upregulating VCL, which inhibited breast cancer metastasis [25]. It has also been reported that the loss of VCL promotes metastasis and predicts poor prognosis in colorectal cancer [26]. Therefore, we speculate that the specific downregulation of VCL in RNMC is associated with the worse prognosis compared with LNMC.

We also observed specific upregulation of RRP12 in RNMC. Kaplan–Meier survival analysis showed that higher RRP12 expression was associated with inferior OS. Integrated bioinformatics analysis revealed RRP12 as a potential gene that could serve as an anticancer target and prognostic marker in CRC [27]. RRP12 overexpression in osteosarcoma cell lines inhibited p53 activity promoted resistance to cytotoxic stress, inhibited apoptosis, and enhanced drug resistance [28]. Whether RRP12 promote RNMC via a similar mechanism remains to be established.

A considerable number of CRC cases develop from colorectal adenoma [29]. Chronic inflammation of the colon is thought to play an important role in this process [30]. Therefore, DEPs in inflammation-related pathways deserve more attention. Compared with NC and LNMC, complement was abnormally activated in RNMC. In the tumor microenvironment, complement plays a dual regulatory role in the occurrence and development of the tumor, affects immune responses, and is also a potential target for tumor immunotherapy [31]. In addition to the complement activation pathway, RNMC-specific DEPs enriched in the MAPK signaling pathway are also associated with inflammation and promote cell proliferation and differentiation [32,33]. Hyperactivation of the MAPK signaling pathway in RNMC not only creates a favorable inflammatory tumor microenvironment for colon cancer progression, but also promotes the proliferation of cancer cells.

RNMC-specific DEPs were also associated with the EMT signaling pathway. In the EMT process, cell–cell and cell-extracellular matrix interactions are remodeled, epithelial cells detach from the basement membrane and are transformed into mesenchymal cells [34]. To some extent, this process is a form of dedifferentiation [35,36], in which tumor cells acquire stem cell characteristics, gain greater tumor-initiating and metastasis potential [37], and develop stronger drug resistance [38]. The EMT pathway is hyperactivated in RNMC, which could explain why RNMC is more prone to recurrence and metastasis.

### 4.2. Correlation between the Expression Profile of Proteins in Tissue and Serum-Derived Extracellular Vesicles in Left-Sided and Right-Sided Colon Cancer

In an analysis of the protein composition of serum-derived extracellular vesicles (EVs) in LNMC and RNMC, Zhong et al. found that LRG1 and SPARC were increased in serum-derived EVs of colon cancer, with a higher abundance in RNMC than in LNMC, indicating that these proteins are potential markers that can be used to predict the prognosis and recurrence of colon cancer [39]. Similar to the expression in serum-derived EVs, we found that SPARC was increased in both LNMC and RNMC tissues (LNMC/NC = 1.811, RNMC/LNMC = 2.139), with slightly higher expression in RNMC than in LNMC (RNMC/LNMC = 1.181). However, in our study, LRG1 expression in colon cancer tissue was lower than that in normal colon tissue (relative abundance LNMC/NC = 0.668, RNMC/NC = 0.839), although the levels in RNMC were still higher relative to LNMC (RNMC/LNMC = 1.257). This may be due to the separation of serum-derived EVs rather than tumor-derived EVs, and the upregulated LRG1 does not necessarily come from the tumor itself. It may also be due to the fact that the tumor secretes EVs containing more LRG1, resulting in a decrease in the local abundance of LRG1. However, further experiments are required to confirm this hypothesis.

In addition, Zhong et al. found that, after stimulation by RNMC serum-derived EVs, the proteins in the EMT pathway of colorectal cancer cells were upregulated, and the invasion and metastasis abilities of the cells were enhanced [39]. We also found that the proteins in the EMT pathway were upregulated in RNMC tissue, which may be responsible for the poorer prognosis of RNMC.

### 4.3. Mucinous Specific DEPs in Right-Sided Colon Cancer Were Mainly Associated with ECM-Related Remodeling, EMT Process, and IL-17 Signal Pathway

Our research found that the mucinous DEPs in right-sided colon cancer were most enriched in the categories of ECM structural constituent, rRNA binding, and ribosome biogenesis. These results implied that ECM-related proteins play more important roles in RMC than in RNMC. Previous studies have revealed that dysregulation of the ECM leads to abnormal behavior and functions of cells and induces cancer metastasis [40]. The cancer-associated ECM is a common feature of a carcinoma and contributes to tumor progression and poor disease outcome [41,42]. Furthermore, ECM proteins promote the formation of invadopodia and lead to tumor cell invasion by driving focal adhesion [43]. Yuzhalin et al. reported that ECM post-translational modifications promote EMT and drive the progression of liver metastasis in mCRC [44]. Recent studies have demonstrated that EMT plays an important role in promoting tumor malignancy and resistance to chemotherapy [45,46]. Combined with our results, it can be inferred that an elevated level of ECM-related proteins are probably related to the poorer prognosis of RMC compared with RNMC.

Of 15 ECM-related proteins, five proteins (FMOD, LUM, BGN, COL14A1, and DCN) were identified in MCODE 1 as hub proteins. We then verified the clinical prognostic value of the five DEPs in an independent TCGA COAD cohort. Two proteins, lumican (LUM) and biglycan (BGN), showed prognostic significance in terms of OS and DFS, respectively. LUM is a small leucine-rich proteoglycan reported to be an important component of the ECM [47]. In accordance with our results, a previous study showed that a high LUM expression was associated with a significantly reduced survival rate in advanced colorectal cancer with nodal metastasis [48]. Furthermore, LUM mRNA overexpression is associated with higher tumor grade, invasion and stage in breast and pancreatic cancers [49,50]. It has been reported that LUM promoted epithelial-to-mesenchymal transition (EMT) in breast cancer and enhanced the migration and invasiveness of colon cancer cells through actin cytoskeletal organization remodeling [51,52,53]. Additionally, LUM may mediate focal contact formation, cytoskeleton remodeling and cell migration by binding to the cancer cell membrane via receptors such as integrins [54]. Zang et al. reported that LUM enhanced cancer progression by targeting the miR200 family to promote EMT, which suggests that LUM is a potential candidate for inhibiting carcinogenic pathways [55]. All these studies indicate that LUM plays critical roles in the progress and migration of right-sided cancers via ECM remodeling. BGN, which is a member of the small leucine-rich proteoglycan family, was also upregulated in the current study. In accordance with our results, several other studies have shown that BGN expression is increased in various cancers including, CRC [56], gastric cancer [57], esophageal cancer [58], and prostate cancer [59]. BGN has also been shown to promote the cancer invasion [60], EMT [61], angiogenesis [60], and chemotherapy resistance [62]. Additionally, BGN was reported to activate the NF-κB pathway by triggering TLR signaling to promote carcinogenesis through loss of immunosuppressive ligands [63,64]. Based on this information, we speculated that these LUM and BGN could be responsible for the poor prognosis in RMC compared with RNMC.

Pathway analysis revealed that the DEPs were enriched in the IL-17 signaling pathway. As shown in Figure 5, most of the downstream proteins in the IL-17 signaling pathway were highly upregulated, indicating that this pathway is strongly associated with tumor progression in RMC. IL-17, which is a potent proinflammatory cytokine, has been shown to promote the growth and metastasis of a wide range of malignancies. Furthermore, a growing body of evidence indicates that the activation of IL-17 signaling induces the expression of inflammatory transcription factors via the NF-κB and MAPK pathways, resulting in cancer formation and progression [65,66,67]. In addition, the IL-17 signal pathway may play a role in remodeling of the stromal architecture of the tumor microenvironment. Among the downstream proteins in the IL-17 pathway, mucin-related upregulated proteins (MUC5AC and MUC5B) have been shown to enhance cell invasion and migration [68]. In this study, we found overexpression of mucin-related proteins in mucinous colon cancers, which supports the reliability of our proteomics data. In addition to mucin-related protein, signal transduction molecules, including S100A8 and S100A9, were also upregulated in our study. These two proteins have been reported to recruit leukocytes by activating the Wnt/β-catenin pathway to stimulate tumor growth in colon cancers [69]. MMPs are an important family of metal-dependent endopeptidases that are responsible for the remodeling of ECM components [70]. MMPs play important roles in the degradation of the ECM to stimulate cancer progression [71]. Matrix metalloproteinase-1 (MMP-1), also known as collagenase-1, has been demonstrated to mediate the pathological progression of diverse cancers [72,73,74]. Wang et al. demonstrated that MMP-1 knockdown suppressed the progression of CRC by inhibiting the PI3K/Akt/c-myc signaling pathway and EMT [75]. All these results indicate that ECM-related EMT and IL-17 signaling pathways are strongly associated with the poor prognosis of right-sided colon cancers. 

### 4.4. Mucinous-Specific DEPs in Left-Sided Colon Cancer Were Mainly Associated with ACE2/Ang-(1–7)/MasR Axis Signal Pathway

For mucinous cancer located in left-sided colon, there was a distinct difference in the DEPs compared with right-sided colon cancer. We next performed GO and KEGG analysis of the DEPs between LMC and LNMC. Interestingly, the DEPs, which were mainly enriched in the renin-angiotensin system, were almost all downregulated in LMN. Thus, we investigated the downregulated DEPs in LMC and performed PPI network to identify the hub proteins. CTSA, CTSG, CPA3, CMA1 and MME all downregulated the ACE2/angiotensin(1–7)/Mas signaling axis, which resulted in the reduction in angiotensin(1–7) expression. As part of the axis induced by ACE2, Ang(1–7) is an endogenous heptapeptide hormone that mediates its biological activity through the Mas-R [76]. Several studies have suggested that Ang(1–7) expression level inhibited tumor proliferation, invasion, and migration in diverse cancers, including nasopharyngeal carcinoma [77], hepatocellular carcinoma [78], prostate cancer [79], and lung cancer [80]. In addition, Yu et al. demonstrated that upregulation of the ACE2/Ang-(1–7)/MasR axis inhibited breast cancer cell metastasis in vivo and in vitro by enhancing store-operated calcium entry [81]. Ang-(1–7) may play an anti-angiogenic role through the attenuation of VEGF and VEGF receptor expression in nasopharyngeal cancer [77]. Taken together, our results indicate that the mucinous specific DEPs showed a strong relationship with ACE2/Ang-(1–7)/MasR axis in left-sided colon cancer. Nonetheless, the underlying mechanism remains to be elucidated.

## 5. Conclusions

In conclusion, we identified dramatic diversity among the proteome profiles of colon cancers in different locations and with distinct histological types. The RNMC-specific DEPs may promote tumor progression and proliferation by activating inflammation-, metastasis-, and proliferation-associated pathways. Further analysis indicated that the increased invasion and metastasis of mucinous colon cancers was mediated through distinct mechanisms in different locations. In right-sided colon cancer, the DEPs in the mucinous type were mainly associated with ECM-related remodeling, EMT process, and the IL-17 signaling pathway. However, in left-sided colon cancer, the mucinous type cancer showed a strong association with the ACE2/Ang-(1–7)/MasR axis in mediating tumor progression and metastasis.

## Data Availability

Data are available to qualified researchers upon reasonable request of the authors.

## Supplementary Materials

The following are available online at https://www.mdpi.com/article/10.3390/curroncol28050305/s1, Table S1: Clinicopathologic parameters of patients, Table S2: All the proteins identified by the MS/MS, Table S3: DEPs between RNMC and LNMC, Table S4: REACTOME pathway analysis of the RNMC-specific upregulated DEPs, Table S5: DEPs between RMC and RNMC, Table S6: The DEPs between LMC and LNMC were grouped into five clusters.
